# Acute Effects of Caffeine and Taurine Co‐Ingestion on Time to Exhaustion and Thermoregulatory Responses to Cycling in the Heat

**DOI:** 10.1002/ejsc.70044

**Published:** 2025-09-16

**Authors:** James Aggett, Joe Page, Jenny Peel, Kevin John, Guilherme Matta, Jamie Tallent, Shane Heffernan, Owen Jeffries, Mark Waldron

**Affiliations:** ^1^ Swansea University Swansea UK; ^2^ Research Institute for Sport and Exercise University of Canberra Canberra Australia; ^3^ School of Sport Exercise and Rehabilitation Sciences University of Essex Colchester UK; ^4^ Department of Physiotherapy Faculty of Medicine Nursing and Health Science School of Primary and Allied Health Care Monash University Victoria Australia; ^5^ Newcastle University Newcastle upon Tyne UK; ^6^ Welsh Institute of Performance Science Swansea University Swansea UK; ^7^ University of the Sunshine Coast Sippy Downs Australia

**Keywords:** endurance, environmental physiology, exercise, nutrition, performance

## Abstract

Caffeine and taurine are commonly co‐ingested pre‐exercise but elicit different thermoregulatory responses; however, their combined effect on thermoregulation is unknown. Therefore, we evaluated the effects of oral caffeine and taurine co‐ingestion on time to exhaustion (TTE) and thermoregulatory responses to cycling in the heat at the gas exchange threshold (GET). Ten healthy nonheat acclimated participants took part in a double‐blind crossover study, completing a TTE in the heat (35°C; 40% relative humidity), cycling at a power output associated with the GET and 1 h after ingesting: caffeine (5 mg/kg) and taurine (50 mg/kg) combined or placebo. Pulmonary gas exchange, core and mean skin temperatures and whole‐body sweat rate (WBSR) were recorded throughout. Heat production was determined using partitional calorimetry. There were no differences in TTE between conditions (*p* = 0.608); however, the rate of oxygen consumption (*p* = 0.017), minute ventilation (*p* = 0.029) and heat production (*p* = 0.019) were higher following the supplement. There were no differences between conditions for skin (*p* = 0.539) and core temperature (*p* = 0.699), mean skin blood flow (*p* = 0.119), respiratory exchange ratio (*p* = 0.546) and WBSR (*p* = 0.897). Pre‐exercise co‐ingestion of caffeine and taurine in the heat had no ergogenic effect despite increasing the ventilatory and metabolic demand. Collectively, these data indicate minimal effects on whole‐body thermoregulation.

## Summary

- Pre‐exercise co‐ingestion of caffeine (5 mg/kg) and taurine (50 mg/kg) did not improve exercise tolerance in the heat *vs*. the placebo condition.
- There was an increased ventilatory and metabolic demand after supplementation but this did not translate to a change in core temperature during exercise.
- Taurine's established vascular effects may have been sufficient to supress the anticipated caffeine‐induced increases in core temperature.
- Further research is needed to clarify how metabolic heat gain was suppressed in the caffeine–taurine condition, which may characterise the role of taurine as part of a multiingredient pre‐exercise supplement in heat environments.

## 1 Introduction

Thermal strain and metabolic demand are increased during prolonged exercise in the heat, which may result in the early cessation of endurance exercise compared to temperate conditions (Galloway and Maughan 1997). To offset metabolic heat gain during exercise, sufficient dry and evaporative heat transfer between the body and the environment is necessary, primarily supported by cutaneous vasodilation and sweating, respectively, thereby attenuating the rate at which core temperature rises (Sawka and Young 2006). Indeed, evaporation of sweat from the skin's surface is a key modifiable heat loss pathway for maintenance of thermal balance during exercise in the heat (Gagge and Gonzales 1996). Metabolic heat gain or dry and evaporative heat loss mechanisms can be modified via training or acclimation/acclimatisation (Ravanelli et al. 2018; Periard et al. 2021). More recent meta‐analytical data have demonstrated that some dietary supplements, such as taurine, can elicit ergogenic effects on performance and reduce core temperature (Peel et al. 2021). In contrast, caffeine supplementation was demonstrated to increase core temperature, despite no performance benefit (Peel et al. 2021).

Taurine has been reported to enhance endurance performance in temperate (Waldron, Knight, et al. 2018) and thermally stressful environments, such as hot and humid conditions (Page et al. 2019; Yu et al. 2024). Its capacity to enhance thermoregulation has been attributed to either the vasodilatory properties of exogenous taurine (Sun et al. 2016) or the increase in sweating that has been reported after single or repeated oral doses (Page et al. 2019; Peel et al. 2024), which could theoretically facilitate heat dissipation through dry or evaporative pathways, respectively. Indeed, both Page et al. (2019) and Peel et al. (2024) reported a decrease in T_core_ following taurine supplementation. Unfortunately, in the only exhaustive trial in the heat, the authors did not report any pulmonary gas exchange measures, such as oxygen uptake, respiratory exchange ratio or minute ventilation (Page et al. 2019). The absence of these measures' limits understanding of how taurine may influence the metabolic and ventilatory responses to exercise in the heat, including the potential effects on substrate utilisation, ventilatory drive or systemic energy cost, which influence heat production.

Caffeine is a widely used stimulant and ergogenic aid for improving endurance performance (Doherty and Smith 2004; Southward et al. 2018) and is recognised by the International Olympic Committee as having strong evidence for providing such benefits (IOC; Maughan et al. 2018). Caffeine increases motivation, alertness (Paluska 2003) and physical performance (Doherty and Smith 2004; Ganio et al., 2009; Goldstein et al. 2010), through its role as an adenosine receptor antagonist (Ribeiro and Sebastião 2010). However, in addition to the meta‐analytical data reported above (Peel et al. 2021), recent studies have demonstrated detrimental effects of ergogenic caffeine doses on thermoregulation, across a range of exercise intensities (Hunt et al. 2021; John et al. 2024). These effects were mechanistically linked to the increase in metabolic heat production (H_prod_) and the potential antagonism of adenosine receptor subtypes (A_2a_) in vascular smooth muscle, thus causing peripheral vasoconstriction (Hein et al. 1999; Khayat and Nayeem 2017). However, mixed results have been reported, with some studies demonstrating beneficial effects of caffeine on endurance performance in the heat (Ping et al. 2010; Pitchford et al. 2014). As recently discussed (John et al. 2024), these conflicting findings could be related to the methods used to control exercise intensity, with exercise domain‐based approaches offering a more suitable solution.

Both caffeine and taurine are of particular interest, since they are frequently consumed in combination as part of energy drinks (Souza et al. 2016), yet given their conflicting thermoregulatory effects, it is not well understood how their co‐ingestion might influence exercise performance and thermophysiological responses in a thermally stressful environment. To date, only one study (Yu et al. 2024) has investigated the effects of caffeine and taurine co‐ingestion in the heat, where a ∼5.7% increase in time to exhaustion (TTE) was reported, without any differences in tympanic temperature. However, a more robust examination of core temperature may be required to identify differences between conditions and the measurement of sweating responses is desirable to understand the potential effects upon evaporative heat transfer.

The aim of the current study was to evaluate the effects of acute oral caffeine and taurine co‐ingestion on TTE at the predetermined thermoneutral gas exchange threshold and thermophysiological responses to exercise in the heat (35°C; 40% relative humidity; RH). It was hypothesised that the supplement would extend TTE in the heat and increase the sweating response to exercise.

## 2 Methods

### 2.1 Participants

Ten healthy, recreationally active and nonheat acclimated adults (males: *n* = 7 and females: *n* = 3) provided written consent to participate in this study (mean ± SD, age 21 ± 1 year; stature 173 ± 9 cm; body mass; 69 ± 9.1 kg and maximal oxygen uptake 3.1 ± 0.8 L/min). A priori sample size was calculated using G*Power (Version 3.1.9.6) based on previously reported changes in TTE performance following acute caffeine supplementation in the heat (Cohen's *d* = 1.02, Ping et al. 2010). In a within‐participants design, a sample of 10 participants was deemed sufficient to identify differences between conditions, with a power of 0.80 and *α* = 0.05. Caffeine habituation status was recorded, 7 participants were classified as habituated and 3 nonhabituated based on the > 100 mg/day criteria (Hunt et al. 2021). The inclusion criteria were as follows: (i) performed regular endurance training (> 150 min/week), (ii) unaffected by any neuromuscular or cardiovascular pathologies and (iii) not taken part in any structured training in the heat within the past year, aged ≥ 18 to < 40 years. The exclusion criteria included (i) self‐reported pregnancy, (ii) history of heat illness, (iii) taking medications that specifically affect body temperature or interfere with thermoregulatory responses and (iv) heat acclimated (visited a hot country in the last 3‐months, sauna use or exercise regularly in hot environments). Participants were recruited from the local university population using posters, email announcements and word of mouth. Recruitment was voluntary and occurred over a 3‐month period prior to data collection and no financial incentives were provided for participation. Testing was conducted in the United Kingdom between October and April. Thus, participants were considered unacclimatised to the heat. Institutional ethical approval was provided for this study, which was conducted in accordance with the Declaration of Helsinki agreement (2018), apart from pretrial registration.

### 2.2 Study Design

This study followed a double‐blind, placebo‐controlled and crossover design. Manual randomisation of trial order and counterbalancing was achieved using a random number generator in Microsoft Excel to designate conditions, with participants sorted by these values to assign half to one condition order (A–B) and half to the reverse (B–A), ensuring balanced order allocation. Supplement encapsulation was completed by a member of the research team who was not involved in data collection, with allocation concealed in sequentially numbered, sealed opaque bags. Blinding efficacy was assessed by asking participants poststudy to guess the order of the supplements they had received. The participants guesses were correct 50% of the time (*n* = 5) and incorrect 50% (*n* = 5) of the time indicating effective blinding. Participants completed three visits at the same time of day, separated by 72–96 h; they refrained from strenuous exercise for 48 h and from alcohol or other supplements for 24 h before each trial. Upon arrival at the laboratory, participants completed a 24 h food diary before the trial, recording portion sizes and food times, ensuring a standardised meal and 500 mL of fluid was consumed 2 h before any exercise trial. This was replicated on each visit to the laboratory and the diaries checked by the research team.

Participants wore cycling shorts, sports socks and training shoes (females also wore a sports bra) and avoided saunas and hot baths throughout the study. On the initial visit, participants underwent familiarisation and preliminary testing—including an incremental ramp test to exhaustion to determine thermoneutral peak oxygen consumption (V̇O_2peak_) and the first gas exchange threshold (GET). Subsequent experimental crossover trials were completed in the heat (35°C, 40% RH): 1 h before each trial, participants ingested either caffeine (5 mg/kg) and taurine (50 mg/kg) or a visually identical placebo (maltodextrin), then performed a time‐to‐exhaustion test at their power output corresponding to the GET, cycling until volitional exhaustion or reaching the withdrawal criterion of 39.5°C core temperature.

### 2.3 Maximal Exercise Test (Visit 1)

Preliminary testing was performed in thermoneutral conditions (∼20°C). Participants were fitted to a cycle ergometer (Monark Exercise AB, Ergomedic 874E, Varberg, Sweden) and completed a warm‐up, comprising a 5‐min cycle at 70 W, followed by a 5‐min rest period before undergoing the test protocol. A fixed cadence of 70 rev/min was sustained, starting at a workload of 70 W, increasing at 21 W/min until volitional exhaustion. Heart rate (HR) was continually monitored (Polar Heart Rate MonitorM400, Warwick, UK). Breath‐by‐breath expired gas was recorded continuously throughout the test using a calibrated analyser (Jaeger Vyntus CPX, Hoechberg, Germany). Gas calibration was performed prior to each trial with known concentrations of gases (15.95% O_2_, 4.97% CO_2_, BAL and N_2_). The turbine transducer was volume‐calibrated using flow rates of 2 L/s and 0.2 L/s (Hans Rudolph, Kansas City, KS). Participants' V̇O_2peak_ was defined as the highest 30 s average across the test. The GET was determined using breath‐by‐breath pulmonary carbon dioxide production (V̇CO_2_) and V̇O_2_ data from the incremental ramp test, using both the simplified v‐slope method (Schneider et al. 1993) and the ventilatory equivalents (Beaver et al. 1986), using the interpretations of two experienced and trained assessors. Power at the GET was adjusted for 2/3 ramp rate and used for the subsequent TTE. The criteria for achieving V̇O_2peak_ was as follows: (i) reaching volitional exhaustion, (ii) unable to maintain cadence > 67 rev/min for more than 10 s and (iii) respiratory exchange ratio > 1.15. A V̇O_2_ plateau was not used, these are often absent or ambiguous in ramp incremental tests. The GET was determined in thermoneutral conditions to increase the accuracy of the breakpoint interpretation as well as facilitating methodological consistency with a number of studies using dietary supplements during exercise in the heat (Page et al. 2019; Fowler et al. 2020; John et al. 2024). We anticipated that this would marginally lower the power output achieved at the GET by approximately 5%, yet produce similar heart rate responses (Bourgois et al. 2023), which was the same for each individual in the current crossover research design.

### 2.4 Experimental Visits (Visits 2 and 3)

Participants arrived at the laboratory at the same time (± 1 h) every visit to ensure consistency between measurements. The experimental trials were conducted in an environmental chamber (JTS Ltd., Brecon, UK; 35 ± 0.1°C, 40 ± 0.3% RH). Urine samples were collected prior to testing to determine hydration status by means of a hand‐held refractometer (Pocket Osmo‐chek, Vitech Scientific Ltd, West Sussex, UK). Values > 600 mOsm/kg/H_2_O indicated the threshold of hypohydration, meaning the participant consumed a further 500 mL of water, before waiting 30 min before testing. Prior to entering the environmental chamber, core temperature (*T*_core_) was determined by means of a rectal thermometer (Walters Medical, W0001 B, England) and data logger (SQ2010; Grant Instruments Ltd., Cambridge, UK), with the probe self‐inserted 10 cm past the anal sphincter. Rectal thermometry, with the probe marked at 10 cm to ensure consistent depth, remains the standard for exercise‐heat studies because it combines participant comfort, low susceptibility to motion artefact and direct comparability with other published data (Page et al. 2019; Fowler et al. 2020; John et al. 2024). It remains widely used and validated as a reliable measure of core temperature (Roberts 1994; Jensen 2000; Casa et al. 2025). The rate of rise in *T*_core_ was determined between the start of the TTE and the time at which a 1.5°C increase in *T*_core_ during the TTE was observed in each participant. This facilitated comparison of participants' *T*_core_ responses across both conditions by accounting for early withdrawal due to reaching the *T*_core_ withdrawal criterion.

Pre‐exercise and postexercise body mass measurements were taken with participants wearing only cycling shorts and the rectal probe (MPMS‐230, Marsden Weighing Group, Oxfordshire, UK) and were used to estimate whole‐body sweat loss. Measurements were taken on calibrated scales with 50 g precision, using identical shorts and probe assembly before and after each trial; postexercise mass was recorded within 5 min of TTE completion, with no fluids consumed between measurements. The shorts and rectal thermometers were weighed before and after measurements and accounted for in the final body mass measurement. Whole‐body sweat rate (WBSR) was calculated by dividing the change in body mass by exercise duration. This method does not distinguish respiratory water loss and may marginally underestimate total fluid loss; however, respiratory evaporation during moderate‐intensity cycling contributes less than 5% of overall water loss (Mitchell et al. 1972; Maughan et al. 2007). Moreover, participants wore a tightly fitted breath‐by‐breath mask that minimised exhaled moisture loss and, owing to the crossover design, any negligible underestimation would be equivalent across conditions. Capillary blood samples were collected 4 minutes postexercise, and blood lactate concentration (B [La]) was measured using an automated analyser (Biosen C_Line, EKF Diagnostic GmbH, Barleben, Germany).

Upon completion of pretesting procedures, participants entered the environmental chamber and rested in a seated position on the ergometer for 5 min. During this period, skin thermistors (Grant Instruments Ltd., Cambridge, UK) were attached to the participant's left side: mid‐calf, mid‐thigh, upper‐chest and mid‐bicep. Skin attachment sites were shaved and cleaned pretest. Core and skin temperature were continuously recorded using a data logger (SQ2010; Grant Instruments Ltd., Cambridge, UK). Weighted mean (T_sk_) was calculated (Ramanathan 1964).

Participants were fitted with a face mask, and pulmonary breath‐by‐breath responses, such as respiratory exchange ratio (RER), minute ventilation (V̇e), V̇O_2_ and V̇CO_2,_ were recorded using the same gas analyser throughout the TTE (Jaeger Vyntus CPX, Hoechberg, Germany). Heart rate (HR) was continually monitored. During the TTE, participants were required to cycle at a power output equivalent to their thermoneutral GET (59% ± 4% V̇O_2peak_; 148 ± 42 W), whilst maintaining a pedal cadence of 70 rev/min. This method of exercise intensity was chosen as it establishes a boundary between moderate and heavy domains (Poole and Jones 2012), which has been used for submaximal assessment of exercise tolerance, whilst sufficiently increasing *H*_prod_ and other thermoregulatory responses (Page et al. 2019; Fowler et al. 2020; John et al. 2024). A member of the research team continuously monitored the participants and provided verbal feedback to maintain the intended cadence within tolerance of the participants' control. The protocol was performed until exhaustion, defined as pedal cadence dropping below 65 rev/min for more than 10 s, or voluntary withdrawal. Thermal comfort (TC) was recorded on a seven‐point scale where − 3 = ‘much too cool’, 0 = ‘comfortable’ and 3 = ‘much too warm’ (Bedford 1936), providing a validated measure of perceptual heat strain, alongside RPE. Rating of perceived exertion (RPE) was measured on a 6‐to‐20‐point Borg scale (Borg 1982), and both were recorded at rest, and every 5 min during the experimental trial and at completion. Upon conclusion of the protocol, skin thermistors were removed, post‐4 min B [La] samples were taken and the participants' postbody mass was recorded.

Whole‐body rates of carbohydrate (CHO) oxidation were calculated using V̇O_2_ and V̇CO_2_ data collected via gas analysis during the TTE. There was no analysis of fat oxidation, owing to the intensity of exercise and the thermally stressful conditions producing RER values > 1 and, therefore, negligible or negative fat oxidation values. The nonprotein RER was used according to Jeukendrup and Wallis 2005):

(1) $$\text{CHO}\;\text{oxidation}\;\left(g/\min\right)=\left[4.210\times \dot{V}{\text{CO}}_{2}\;\left(L/\min\right)\right]-\;\left[2.962\times \dot{V}O_{2}\;\left(L/\min\right)\right].$$

### 2.5 Partitional Calorimetry

Participants *H*_prod_ during the TTE was determined by subtracting the rate of mechanical work (Wk) from the rate of metabolic energy expenditure (*M*):

(2) $$H_{\text{prod}}=M\;–\;\text{Wk}\;\left[W\right],$$

where metabolic energy expenditure (M) was determined using measured V̇O_2_ (L/min) and RER in the 10% epoch of each TTE:

(3) $$M=\dot{V}O_{2\;x\;}\frac{\left((\frac{\text{RER}-0.7}{0.3})\;x\;21.13\right)+\left((\frac{1.0-\text{RER}}{0.3})\;x\;19.62\right)}{60}x\;1000\;\left[W\right].$$

The *H*_prod_ (W/m^2^) was expressed relative to the participants' body surface area (Cramer and Jay 2014):

(4) $$H_{\text{prod}}=\frac{H_{\text{prod}}}{\text{BSA}}\left[W/m^{2}\right]$$

Du bois and Du bois equation [Du Bois and Du Bois 1916]:

(5) $$\text{BSA}=0.00718\;x\;(\text{body}\;\text{mass}\;\left({\text{kg})}^{0.425}\right)\;x\;(\text{height}\;\left({\text{cm})}^{0.725}\right)\;\left[m^{2}\right].$$

On the assumption that blood entering and leaving the cutaneous circulation was equal to core and skin temperatures, respectively, estimated skin blood flow (SkBF) in the 10% epoch of each TTE was determined as follows (Sawka and Young 2006):

(6) $$\text{SkBF}=\frac{\left(\frac{1}{\text{SH}}\;\cdot \;H_{\text{prod}}\right)}{\left(T_{\text{core}-}T_{\text{skin}}\right)}.$$

where SH = specific heat of the blood (∼1 kcal/°C) and *H*_prod_ is expressed in kcal/min. *T*_core_ and *T*_skin_ were taken as the mean measurements in the 10% epoch of each TTE to provide an estimated SkBF measure.

### 2.6 Supplementation

All supplements were acquired in white anhydrous powder form and were separated into gelatine capsules using analytical balance scales (Ohaus, Navigator N24120, Nänikon Switzerland; resolution 0.01 g). The participants' body mass was recorded during visit 1 was subsequently used to measure correct doses, such that supplements were balanced and an equal number of capsules were ingested by the participants between visits 2 and 3. The capsules contained either caffeine and taurine (C + T; caffeine 5 mg/kg body mass; Blackburn Distributions Ltd., Burnley, UK; taurine 50 mg/kg body mass; My Protein, Manchester, UK) or a placebo (Maltodextrin, My Protein, Manchester, UK) and were administered 1 h prior to exercise. The dose and timing of the supplement was based on previous recommendations when co‐ingesting these supplements (Warnock et al. 2017; Jeffries et al. 2018). The 1‐h period prior to exercise aligns approximately with peak plasma availability of caffeine (Arnaud and Welsch 1982) and taurine (Ghandforoush‐Sattari et al. 2010), with tolerance for some individual variability in response.

### 2.7 Statistical Analysis

The normality of the residuals was assessed using the Shapiro–Wilk test, after which two‐way analyses of variance were used to determine the effect of condition (C + T vs. placebo) and time (10%–100% epochs) on V̇O_2_, V̇e, RER, CHO oxidation rate, *H*_prod_, SkBF, *T*_core_ and *T*_skin_. Two‐tailed paired samples *t*‐tests were used to compare conditions for the primary outcome performance measure (TTE) as well as post‐exercise B [La], WBSR and the rate of rise in *T*_core_. To account for the discontinuous measurement of TC, RPE and HR, data were measured in quartiles across the trial. A Greenhouse–Geisser correction was applied when the assumption of sphericity was violated. Significant main and interaction effects were analysed using Bonferroni‐corrected *post hoc* tests. The TC analysis demonstrated consistent abnormal distribution; therefore, analysis was conducted using the nonparametric aligned ranks test for repeated measures (‘*ARTool’* package in RStudio). Statistical significance was set at *p* ≤ 0.05. Besides the nonparametric tests, all data collected were analysed using IBM SPSS Statistics (28.0.1.1; SPSS Inc., Chicago, Illinois, USA). Partial eta‐squared (*η* ^*2*^ *p*) was reported to calculate the magnitude of effect according to the following criteria: 0.02, a small difference; 0.13, a moderate difference and 0.26, a large difference (Cohen 1988). Cohen's *d* for repeated measures was calculated as the mean difference divided by the standard deviation of the differences to interpret the effect of pairwise changes. Cohen threshold effect sizes were classified as small (*d* = 0.2), medium (*d* = 0.5) and large (*d* ≥ 0.8).

## 3 Results

### 3.1 Time to Exhaustion Analysis

**FIGURE 1:**
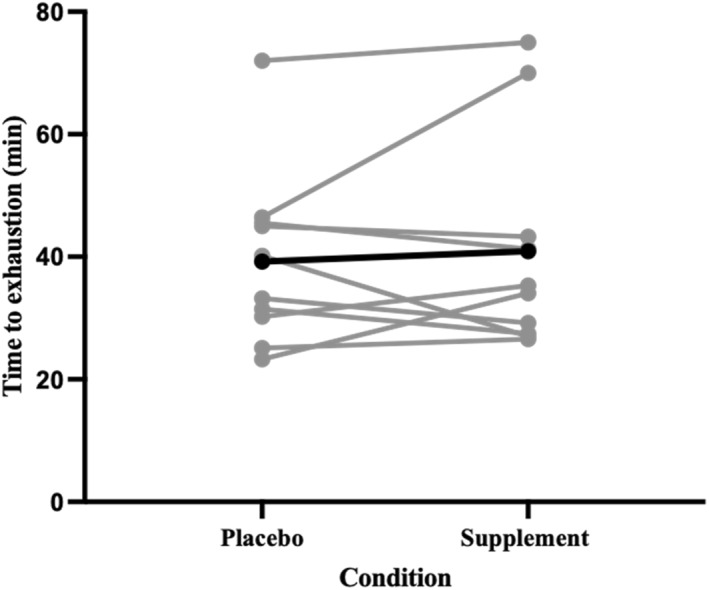
Time to exhaustion at the gas exchange threshold following a co‐ingestion of caffeine and taurine or placebo (*n* = 10) in a hot environment (35°C, 40% RH). Black bar denotes mean response.

There were no differences between conditions for TTE when cycling at the power output associated with ventilatory threshold in 35°C/40% RH (placebo = 39.23 ± 14.27 min and C + *T* = 40.92 ± 17.66 min) (*t*_(9)_ = −0.531, *p* = 0.304, *d* = 0.1 and Figure 1).

### 3.2 Cardiometabolic Responses

**FIGURE 2:**
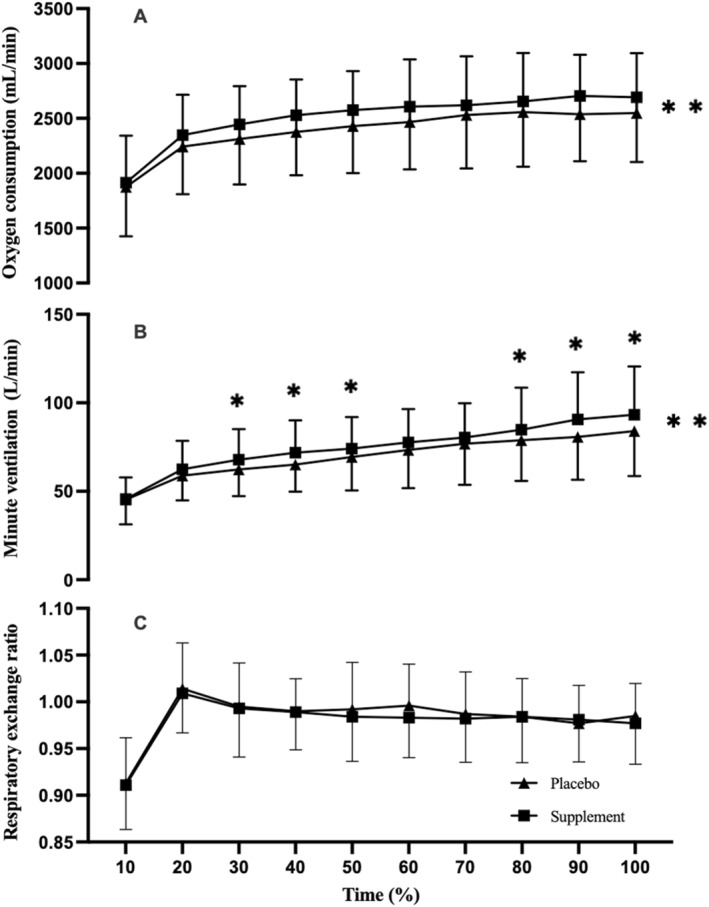
Oxygen consumption (A), minute ventilation (B) and respiratory exchange ratio (C) at the gas exchange threshold following a co‐ingestion of caffeine and taurine or placebo (*n* = 10) in a hot environment (35°C, 40% RH). * symbolises significant interaction effect and ** symbolises significant condition effect denoting higher in C + T (*p <* 0.05).

**FIGURE 3:**
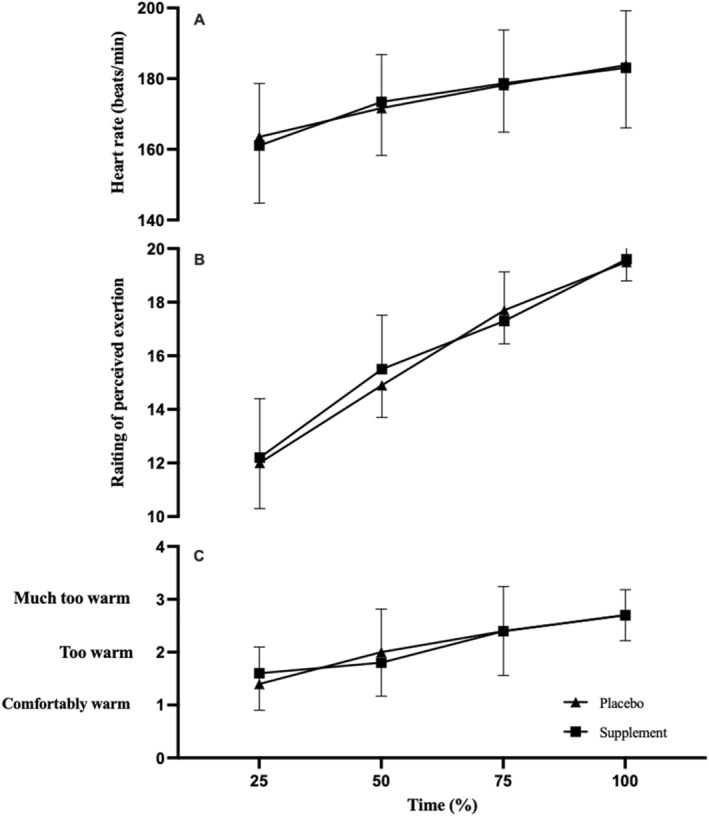
Heart rate (A), rating of perceived exertion (B) and thermal comfort (C) at the gas exchange threshold following a co‐ingestion of caffeine and taurine or placebo (*n* = 10) in a hot environment (35°C, 40% RH).

There were main condition effects for V̇O_2_ (*F*_(1,9)_ = 8.440, *p* = 0.017 and *η* ^*2*^ *p* = 0.484; Figure 2A) and V̇e (*F*_(1,9)_ = 6.688, *p* = 0.029 and *η* ^*2*^ *p* = 0.426; Figure 2B) but no main effects on RER (*F*_(1,9)_ = 0.394, *p* = 0.546 and *η* ^*2*^ *p* = 0.042; Figure 2C) and HR (*F*_(1,9)_ = 0.031, *p* = 0.864 and *η* ^*2*^ *p* = 0.003; Figure 3A). There were no interaction effects for V̇O_2_ (*F*_(1,9)_ = 0.996, *p* = 0.450 and *η* ^*2*^ *p* = 0.100), RER (*F*_(1,9)_ = 0.487, *p* > 0.693 and *η* ^*2*^ *p* = 0.051) and HR (*F*_(1,9)_ = 0.902, *p* = 0.430 and *η* ^*2*^ *p* = 0.082); however, there were interaction effects for V̇e (*F*_(1,9)_ = 2.704, *p* = 0.008 and *η* ^*2*^ *p* = 0.231). Pairwise analysis demonstrated that V̇e was higher at the 30 (*d* = 0.745), 40 (*d* = 0.763), 50 (*d* = 0.803), 80 (*d* = 1.071), 90 (*d* = 1.025) and 100% (*d* = 0.785) epochs in the C + T condition (*p* < 0.05). There were no main effects of C + T (*F*_(1,9)_ = 0.074, *p* = 0.792 and *η* ^*2*^ *p* = 0.008) or time interaction (*p* > 0.05) on CHO oxidation. The mean CHO oxidation for C + T versus placebo was 3.1 ± 0.9 g/min versus 3.2 ± 1.0 g/min, respectively.

### 3.3 Thermoregulatory Responses

**FIGURE 4:**
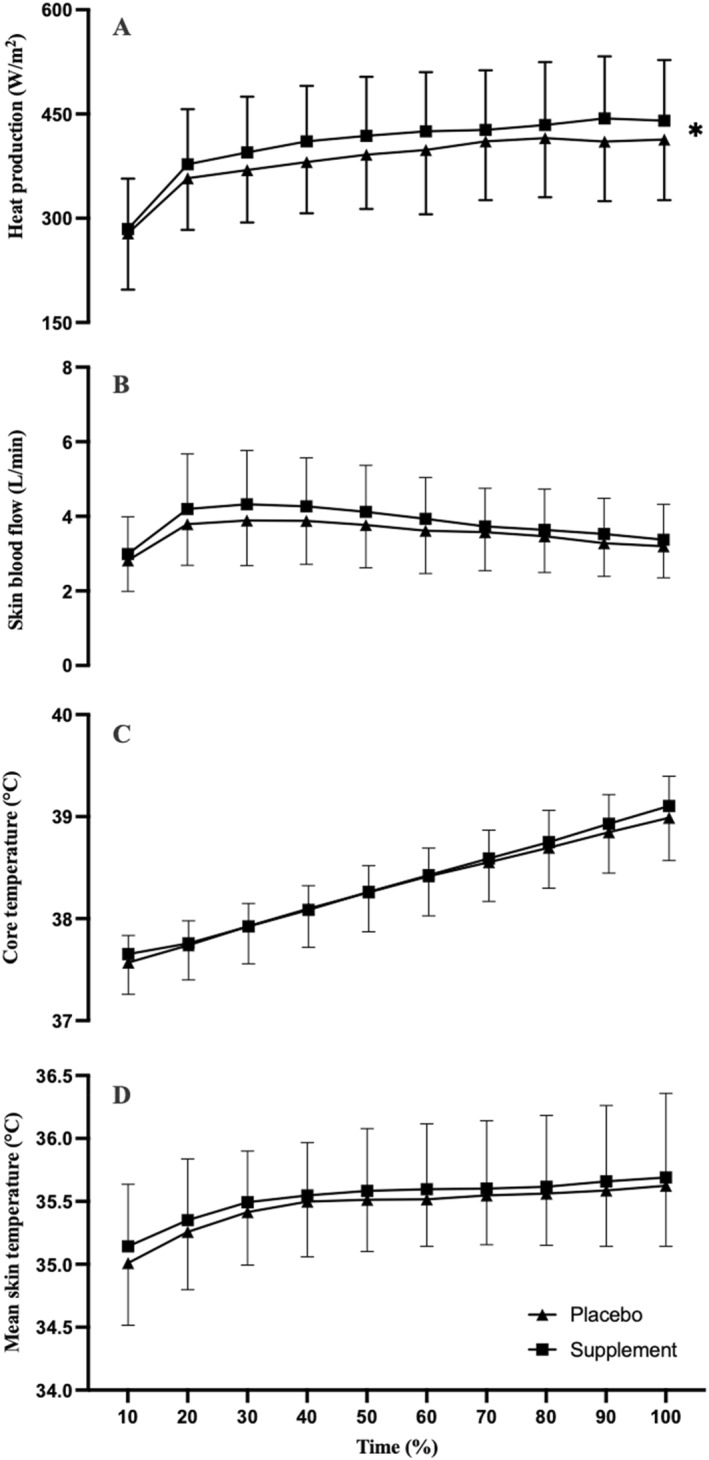
Heat production (A), skin blood flow (B), core temperature (C) and mean skin temperature (D) at the gas exchange threshold following a co‐ingestion of caffeine and taurine or placebo (*n* = 10) in a hot environment (35°C, 40% RH). * symbolises significant condition effect denoting higher in C + T (*p <* 0.05).

**FIGURE 5:**
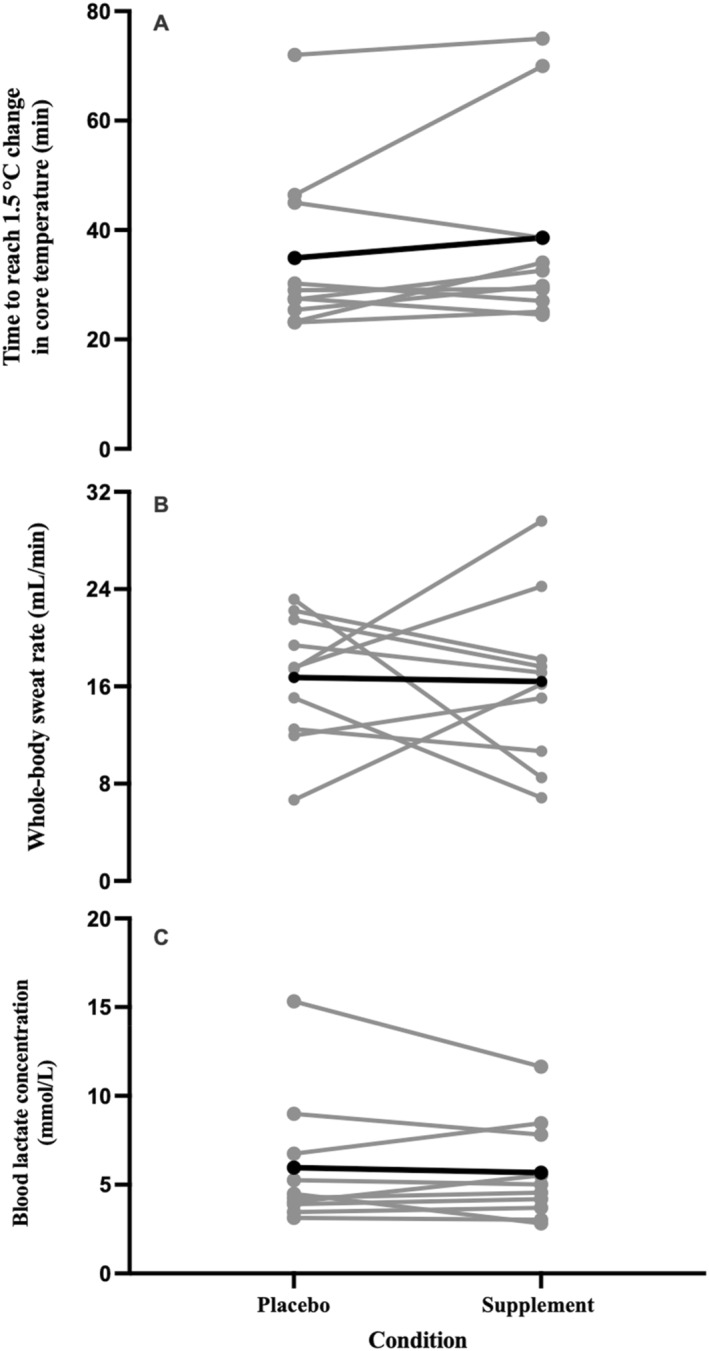
Time to reach 1.5°C change in core temperature (A), whole‐body sweat rate (B) and blood lactate concentration (C) response at the gas exchange threshold following a co‐ingestion of caffeine and taurine or placebo (*n* = 10) in a hot environment (35°C, 40% RH). Black bar denotes mean response.

There were condition effects for *H*_prod_ (*F*_(1,9)_ = 8.129, *p* = 0.019 and *η* ^*2*^ *p* = 0.475; Figure 4A) but not for SkBF (*F*_(1,9)_ = 2.971, *p* = 0.119 and *η* ^*2*^ *p* = 0.248; Figure 4B), *T*_core_ (*F*_(1,9)_ = 0.159, *p* = 0.699 and *η* ^*2*^ *p* = 0.017; Figure 4C), *T*_skin_ (*F*_(1,9)_ = 0.409, *p* = 0.539 and *η* ^*2*^ *p* = 0.043; Figure 4D), WBSR (*t*_(9)_ = 0.133, *p* = 0.449 and *d* = 0.07; Figure 5B) and post B [La] (*t*_(9)_ = 0.558, *p* = 0.295 and *d* = 0.08; Figure 4C). There were no interaction effects for *H*_prod_ (*F*_(1,9)_ = 1.041, *p* = 0.388 and *η* ^*2*^ *p* = 0.104), SkBF (*F*_(1,9)_ = 0.473, *p* = 0.666 and *η* ^*2*^ *p* = 0.050), *T*_core_ (*F*_(1,9)_ = 1.274, *p* = 0.264 and *η* ^*2*^ *p* = 0.124) and *T*_skin_ (*F*_(1,9)_ = 0.097, *p* = 0.895 and *η* ^*2*^ *p* = 0.011). There were also no differences between conditions for time to reach a 1.5°C change from *T*_core_ baseline (*t*_(9)_ = − 1.355, *p* = 0.104 and *d* = 0.2; Figure 3A).

### 3.4 Perceptual Responses

There was no main effect of condition on TC (*F*_(1,3)_ = 0.637 and *p* = 0.428; Figure 3C) or RPE (*F*_(1,9)_ = 0.175, *p* = 0.685; Figure 3B). TC increased with time across both conditions (*F*_(1,3)_ = 51.515 and *p* < 0.01) but there were no interaction effects (*F*_(1,3)_ = 0.981 and *p* = 0.407). Similarly, there were no interaction effects on TC (*F*_(1,3)_ = 0.981 and *p* = 0.407) or RPE (*F*_(1,9)_ = 1.681 and *p* = 0.212).

## 4 Discussion

The current study evaluated the effects of acute caffeine (5 mg/kg) and taurine (50 mg/kg) co‐ingestion on TTE and thermoregulatory responses to cycling in the heat. Contrary to the proposed hypothesis, there was no ergogenic effect following pre‐exercise co‐ingestion of C + T in the heat. However, compared to placebo, ingesting C + T 60 min before exercise resulted in increased V̇O_2_, V̇e and *H*_prod_, which was sustained for the majority of the TTE. Interestingly, despite these effects, there were no changes in core and skin temperature or sweating between conditions, thus indicating minimal impact on thermoregulation. It has been reported that isolated taurine supplementation in thermoneutral and thermally stressful environments enhances thermoregulatory responses (Page et al. 2019; Waldron, Patterson, et al. 2018; Yu et al. 2024). Similarly, isolated caffeine supplementation in temperate environments has endurance benefits (Doherty and Smith 2004; Southward et al. 2018), although there are mixed findings in hot environments (John et al. 2024; Ping et al. 2010; Pitchford et al. 2014; Suvi et al. 2017). Therefore, the negligible effects of C + T on endurance performance, alongside a number of physiological measures, such as WBSR, HR, B [La] and skin and core temperatures, indicates that co‐ingestion of these supplements at previously reported ergogenic doses has minimal effects when ingested in the heat.

A novel finding of the current study was the increased V̇O_2_ and V̇e following C + T. Besides studies of energy drinks, where lower C + T doses are consumed (Candow et al. 2009; Astorino et al. 2011; Del Coso et al. 2013; Hoyte et al. 2013), no other study has reported cardiometabolic responses, such as V̇O_2_, V̇e, RER and substrate utilisation, when co‐ingesting C + T. Herein, the notable increase in V̇O_2_ (∼5.4%), despite the fixed‐workload exercise design, was sufficient to drive metabolic heat production ∼6.2% higher in the C + T condition. The increase in V̇O_2_ was accompanied by no change in RER, indicating an equivalence in fuel utilisation. Although taurine (Rutherford et al. 2010; Simmonds et al. 2020) and caffeine (Collado‐Mateo et al. 2020) have both been reported to affect metabolic efficiency during exercise via increases in fat oxidation, these findings have not been reported elsewhere (Galloway et al. 2008; Hodgson et al. 2013; John et al. 2024), which was also the case in the current study. This suggests that the mechanisms by which C + T influences metabolic responses might be independent of changes in substrate utilisation.

Recently, it has been reported that caffeine supplementation drives *H*_prod_ through increases in V̇O_2_ (∼7.9%), causing a significant increase in *T*_core_ when exercising in the heat (John et al. 2024). These results are somewhat similar to that observed in the current study, suggesting that the effect of caffeine on these pulmonary measures is sustained when co‐ingested with taurine. Increases in V̇e of a similar magnitude have been reported during exercise following ingestion of caffeine at similar doses (Chapman and Stager 2008; Powers et al. 1986). It is likely that caffeine's (a methylxanthine and theophylline) effects on ventilatory drive, either via an increase in diaphragm contractility (Supinski et al. 1984) or an antagonistic effect on adenosine receptor sub‐types (A2_b_; Feoktistov et al. 1998), were observed in the TTE, which may have induced secondary increases in whole‐body V̇O_2_ (Aaron et al. 1992) and thus *H*_prod_. The rapid absorption of caffeine postsupplementation, with average peak bioavailability achieved at 1 h (Blanchard and Sawers 1983), may explain the early onset of increased V̇e and V̇O_2_ in the TTE. It is interesting that the reduction in gross efficiency, denoted by the increased V̇O_2_ despite the same mechanical workload in the C + T condition, did not alter the TTE result. Indeed, the lack of influence upon measures of body temperature post C + T supplementation is at odds with the increased metabolic demand and could be attributed to the co‐ingestion with taurine, which appears to have opposing thermoregulatory and ergogenic effects in the heat (Peel et al. 2024; Page et al. 2019). Although the combined ingestion of C + T may have influenced the thermoregulatory responses observed, the absence of caffeine‐only and taurine‐only control conditions prevents any firm conclusions about the individual contribution of each supplement.

There were no significant differences in final *T*_core_ and *T*_skin_, which is consistent with another study (Yu et al. 2024). This occurred despite greater *H*_prod_ in the C + T condition, alongside no differences in SkBF. Thus, there was no indication of changes in dry heat losses (i.e., convective, radiative and conductive). Isolated caffeine has been reported to increase *H*_prod_ and *T*_core_, alongside reductions in SkBF (Hunt et al. 2021; John et al. 2024). Thus, the introduction of taurine to the caffeine supplement appears to have altered the estimation of SkBF despite similar effects on *H*_prod_. The potential vasoactive property of taurine in the mixed supplement may have counteracted the constrictive properties of caffeine and provides one potential explanation for the similarity in SkBF, and therefore *T*_core_, between conditions. It was also anticipated that WBSR would be increased, given reports that both caffeine and taurine can drive the sweating response when ingested alone (Page et al. 2019; John et al. 2024; Peel et al. 2024), and that increases in sweating are a natural consequence of the observed increase in *H*_prod_ (Peel et al. 2021). However, other studies have reported no change in sweating after caffeine ingestion (Del Coso et al. 2009; Ganio et al., 2010; Gonzalez et al. 2020; Hunt et al. 2021). It is possible that small differences in the pharmacokinetics of caffeine and taurine, with earlier bioavailability of caffeine dominating the response (Arnaud and Welsch 1982; Ghandforoush‐Sattari et al. 2010), meant that the total effects of taurine were not realised across the shorter TTE. Unfortunately, we do not have the pharmacokinetic data to support this assertion. Therefore, we are uncertain why there was no difference in the sweating response, and further research is warranted to understand the mechanistic underpinnings for the unanticipated outcome when the supplements were co‐ingested. Given our previous reports on the thermoregulatory effects of isolated taurine or caffeine in the heat when adopting the same exercise protocol (Page et al. 2019; John et al. 2024), coupled with the clear indication of thermal strain among participants in the current study, we are confident that sufficient thermal stress was applied. However, with the current time‐to‐exhaustion trials averaging approximately 40 min, it is possible that the cumulative metabolic and thermoregulatory responses that may emerge during longer‐duration endurance exercise were not captured. Future studies utilising extended protocols (e.g., > 60 min) are warranted to determine whether prolonged exposure unmasks more pronounced effects of caffeine and taurine on performance and heat stress.

The supplement elicited minimal changes in B [La]. Although Yu et al. (2024) reported that co‐ingesting C + T caused increases in B [La] in comparison to a placebo as well as both isolated C + T conditions, this occurred as a result of increased exercise time (i.e., improvements in the TTE trial). However, the lack of difference in B [La] between conditions herein is inconsistent with the suggestions that taurine can elicit changes in substrate metabolism, with reductions in glycolytic metabolism contribution or enhanced fat oxidation (De Carvalho et al. 2017; Simmonds et al. 2020; Rutherford et al. 2010). These results are equivocal as there has been no evidence of changes in substrate metabolism during low intensity walking exercise in the heat (Peel et al. 2024), despite previous findings of reduced post‐exercise B [La] at higher intensities in a hot environment (Page et al. 2019). Similarly, caffeine supplementation did not affect substrate metabolism during exercise of the same intensity used in the current study, in the heat, compared to a placebo (John et al. 2024). However, some studies have reported increased B [La] despite no performance differences, following caffeine ingestion compared to a placebo (Suvi et al. 2017). Therefore, there is a lack of consensus regarding the effects of isolated caffeine or taurine on B [La] or substrate metabolism during exercise in the heat. Nevertheless, when supplemented in combination, we report that C + T caused no change in these responses.

In accordance with the TTE results, where we reported no differences between conditions, both RPE and TC followed the same time course and magnitude irrespective of the ingested supplement. The similarity in the physiological responses between conditions, denoted by HR, B [La], WBSR, *T*_core_ and *T*_skin_ results, explains the lack of effect upon the participants' perceptual scores. Thus, there appears to be no thermoregulatory, performance or perceptual benefit of C + T supplementation (at the doses used) during exercise in the heat. Consequently, there would be no apparent benefit for athletes to co‐ingest C + T prior to exercise in such conditions. However, there is potential to accelerate the progression of exercise‐induced hyperthermia with prior caffeine administration (Peel et al. 2021), which increases the risk of heat illness if unrecognised or untreated. There was one participant who appeared to respond more favourably to the supplement in the TTE, with a notable increase in their TTE of ∼20 min. Physiological data were consistent with those observed in recreationally trained individuals and the participant met the criteria to remain in the study, but it seems unlikely that the supplement enhanced performance by this magnitude. Nevertheless, further analysis is needed to understand the lack of consistency in TTE response across all participants and the potential for supplement ‘responders’. This was not the aim of the current study, which limits the ability to conduct further analysis. Participants included both habitual and nonhabitual caffeine consumers, which may have attenuated the stimulatory response in habituated individuals and exaggerated it in nonhabituated users. However, no clear effect or pattern was observed based on habitual consumption, aligning with findings from previous studies (Gonçalves et al. 2017; Clarke and Richardson 2020), thereby suggesting that habituation did not meaningfully influence performance outcomes.

Although there are many ways to control exercise intensity in thermally stressful conditions, utilisation of the power at GET enables the domain‐specific prescription of exercise intensity, which is not possible when prescribing intensity based on alternative methods, such as fractions of $\dot{V}$ O_2peak_ (Mann et al. 2013). Although the GET is inevitably altered by the hot environmental conditions, this approach ensures equivalent baseline conditions, the advantages of which have previously been discussed for studies such as the current investigation (John et al. 2024). Herein, we determined GET in thermoneutral conditions to ensure consistent identification of the first metabolic threshold, independent of ventilatory and cardiovascular alterations induced by acute thermal stress. This approach mirrors other supplement intervention studies examining thermoregulatory responses (Page et al. 2019; Fowler et al. 2020; John et al. 2024). Conducting the test in a hot environment would also likely impair maximal effort due to early thermal discomfort or fatigue, particularly in nonheat‐acclimated individuals, thereby compromising the accuracy of GET detection as well as $\dot{V}$ O_2peak_. Consequently, prescribing thermoneutral GET‐derived power in 35°C ensured precise within‐ and between‐study comparisons but caused participants to rapidly enter the heavy‐to‐severe intensity domains reflected by RER > 1.0 and B [La] > 4 mmol/L. This contributed to the shortened TTE (approximately 20–40 min). Although the GET‐based fixed‐intensity protocol provided control of metabolic and thermal load across trials, it does not replicate the intermittent demands of most sports. However, it does enable direct comparison with previous studies. To prolong the work done during trials, future studies could attempt to apply heat‐adjusted threshold intensities, which may help to further characterise the capacity of these supplements to affect thermoregulation. Investigation of taurine or caffeine and taurine co‐ingestion in real‐world sports settings would also be worthwhile. Finally, closer control of pre‐exercise nutrition may also be required as the use of food diaries can influence participants' habitual eating behaviours or inaccurately report their dietary intake.

## 5 Conclusion

The current study observed no ergogenic or thermoregulatory benefit of C + T when cycling in the heat at an intensity equivalent to the GET. However, there were increases in V̇O_2_, V̇e and *H*_prod_, which demonstrate a thermogenic effect of the supplement. Despite this, there were no changes in *T*_core_, *T*_skin_, estimated SkBF or sweating between conditions, thus indicating minimal effects on whole‐body thermoregulation. Interpretation of the current data, alongside that from previous studies, indicates that the inclusion of caffeine in the mixed supplement resulted in changes in parameters of pulmonary gas exchange, but it is unclear why this did not translate to any performance, thermophysiological or perceptual effects. Given the established roles of taurine in the peripheral vasculature (Sun et al. 2016; Ulusoy et al. 2017), its inclusion in the supplement may have been sufficient to supress the anticipated effects of caffeine on *T*_core_. Further work is required to mechanistically understand how metabolic thermal gain was supressed in the C + T condition.

## Conflicts of Interest

The authors declare no conflicts of interest.

## Data Availability

All data are available upon reasonable request.

## References

[ejsc70044-bib-0001] Aaron, E. A. , K. C. Seow , B. D. Johnson , and J. A. Dempsey . 1992. “Oxygen Cost of Exercise Hyperpnea: Implications for Performance.” Journal of Applied Physiology 72, no. 5: 1818–1825. 10.1152/jappl.1992.72.5.1818. 1601791

[ejsc70044-bib-0002] Arnaud, M. J. , and C. Welsch . 1982. “Theophylline and Caffeine Metabolism in Man.” in Theophylline and other Methylxanthines/Theophyllin und andere Methylxanthine: Proceedings of the 4th International Symposium, Frankfurt/M., 29th and 30th May, 1981/Vorträge des 4. Internationalen Symposiums, Frankfurt/M., 29. und 30. Mai, 1981, 135–148.

[ejsc70044-bib-0003] Astorino, T. A. , A. J. Matera , J. Basinger , M. Evans , T. Schurman , and R. Marquez . 2011. “Effects of Red Bull Energy Drink on Repeated Sprint Performance in Women Athletes.” Amino Acids 42, no. 5: 1803–1808. 10.1007/s00726-011-0900-8. 21461905

[ejsc70044-bib-0004] Beaver, W. L. , K. Wasserman , and B. J. Whipp . 1986. “A New Method for Detecting Anaerobic Threshold by Gas Exchange.” Journal of Applied Physiology 60, no. 6: 2020–2027. 10.1152/jappl.1986.60.6.2020. 3087938

[ejsc70044-bib-0005] Bedford, T. 1936. “The Warmth Factor in Comfort at Work: A Physiological Study of Heating and Ventilation.” In Industrial Health Research Board. 76th ed. HMSO.

[ejsc70044-bib-0006] Blanchard, J. , and S. J. A. Sawers . 1983. “The Absolute Bioavailability of Caffeine in Man.” European Journal of Clinical Pharmacology 24, no. 1: 93–98. 10.1007/bf00613933. 6832208

[ejsc70044-bib-0007] Borg, G. A. 1982. “Psychophysical Bases of Perceived Exertion.” Medicine & Science in Sports & Exercise 14, no. 5: 377–381. 10.1249/00005768-198205000-00012. 7154893

[ejsc70044-bib-0008] Bourgois, G. , A. L. Colosio , K. Caen , J. G. Bourgois , P. Mucci , and J. Boone . 2023. “The Effect of Acute Heat Exposure on the Determination of Exercise Thresholds From Ramp and Step Incremental Exercise.” European Journal of Applied Physiology 123, no. 4 (April): 847–856. 10.1007/s00421-022-05106-y. 36507952

[ejsc70044-bib-0009] Candow, D. G. , A. K. Kleisinger , S. Grenier , and K. D. Dorsch . 2009. “Effect of Sugar‐Free Red Bull Energy Drink on High‐Intensity Run Time‐to‐Exhaustion in Young Adults.” Journal of Strength & Conditioning Research 23, no. 4: 1271–1275. 10.1519/jsc.0b013e3181a026c2. 19528841

[ejsc70044-bib-0010] Casa, D. J. , S. M. Becker , M. S. Ganio , et al. 2025. “Validity of Devices That Assess Body Temperature During Outdoor Exercise in the Heat.” Journal of Athletic Training 42, no. 3: 333–342. PMC1978469 18059987

[ejsc70044-bib-0011] Chapman, R. F. , and J. M. Stager . 2008. “Caffeine Stimulates Ventilation in Athletes With Exercise‐Induced Hypoxemia.” Medicine & Science in Sports & Exercise 40, no. 6: 1080–1086. 10.1249/mss.0b013e3181667421. 18460998

[ejsc70044-bib-0012] Clarke, N. D. , and D. L. Richardson . 2020. “Habitual Caffeine Consumption Does Not Affect the Ergogenicity of Coffee Ingestion During a 5 Km Cycling Time Trial.” International Journal of Sport Nutrition and Exercise Metabolism 31, no. 1: 1–8. 10.1123/ijsnem.2020-0204. 33260141

[ejsc70044-bib-0013] Cohen, J. 1988. Statistical Power Analysis for the Behavioral Sciences. 2nd ed. Routledge.

[ejsc70044-bib-0014] Collado‐Mateo, D. , A. M. Lavín‐Pérez , E. Merellano‐Navarro , and J. D. Coso . 2020. “Effect of Acute Caffeine Intake on the Fat Oxidation Rate During Exercise: A Systematic Review and Meta‐Analysis.” Nutrients 12, no. 12: 3603. 10.3390/nu12123603. 33255240 PMC7760526

[ejsc70044-bib-0015] Cramer, M. N. , and O. Jay . 2014. “Selecting the Correct Exercise Intensity for Unbiased Comparisons of Thermoregulatory Responses Between Groups of Different Mass and Surface Area.” Journal of Applied Physiology 116, no. 9: 1123–1132. 10.1152/japplphysiol.01312.2013. 24505102

[ejsc70044-bib-0016] De Carvalho, F. G. , R. A. Barbieri , M. B. Carvalho , et al. 2017. “Taurine Supplementation Can Increase Lipolysis and Affect the Contribution of Energy Systems During Front Crawl Maximal Effort.” Amino Acids 50, no. 1: 189–198. 10.1007/s00726-017-2505-3. 29082444

[ejsc70044-bib-0017] Del Coso, J. , E. Estevez , and R. Mora‐Rodriguez . 2009. “Caffeine During Exercise in the Heat.” Medicine & Science in Sports & Exercise 41, no. 1: 164–173. 10.1249/mss.0b013e318184f45e. 19092693

[ejsc70044-bib-0018] Del Coso, J. , J. Portillo , G. Muñoz , J. Abián‐Vicén , C. Gonzalez‐Millán , and J. Muñoz‐Guerra . 2013. “Caffeine‐Containing Energy Drink Improves Sprint Performance During an International Rugby Sevens Competition.” Amino Acids 44, no. 6: 1511–1519. 10.1007/s00726-013-1473-5. 23462927

[ejsc70044-bib-0019] Doherty, M. , and P. M. Smith . 2004. “Effects of Caffeine Ingestion on Exercise Testing: A Meta‐Analysis.” International Journal of Sport Nutrition and Exercise Metabolism 14, no. 6: 626–646. 10.1123/ijsnem.14.6.626. 15657469

[ejsc70044-bib-0020] Du Bois, D. 1916. “Clinical Calorimetry.” Archives of Internal Medicine XVII, no. 6_2: 863. 10.1001/archinte.1916.00080130010002.

[ejsc70044-bib-0021] Feoktistov, I. , I. Biaggioni , R. Polosa , and S. T. Holgate . 1998. “Adenosine A2B Receptors: A Novel Therapeutic Target in Asthma?” Trends in Pharmacological Sciences 19, no. 4: 148–153. 10.1016/s0165-6147(98)01179-1. 9612090

[ejsc70044-bib-0022] Fowler, R. , O. Jeffries , J. Tallent , et al. 2020. “No Thermoregulatory or Ergogenic Effect of Dietary Nitrate Among Physically Inactive Males, Exercising Above Gas Exchange Threshold in Hot and Dry Conditions.” European Journal of Sport Science 21, no. 3: 370–378. 10.1080/17461391.2020.1739144. 32130090

[ejsc70044-bib-0023] Gagge, A. P. , and R. R. Gonzales . 1996. “Mechanisms of Heat Exchange.” In Handbook of Physiology Section 4 Environmental Physiology, edited by M. J. Fregley and C. M. Blatteis , 45–48. Oxford University Press.

[ejsc70044-bib-0024] Galloway, S. D. R. , and R. J. Maughan . 1997. “Effects of Ambient Temperature on the Capacity to Perform Prolonged Cycle Exercise in Man.” Medicine & Science in Sports & Exercise 29, no. 9: 1240–1249. 10.1097/00005768-199709000-00018. 9309637

[ejsc70044-bib-0025] Galloway, S. D. R. , J. L. Talanian , A. K. Shoveller , G. J. F. Heigenhauser , and L. L. Spriet . 2008. “Seven Days of Oral Taurine Supplementation Does Not Increase Muscle Taurine Content or Alter Substrate Metabolism During Prolonged Exercise in Humans.” Journal of Applied Physiology 105, no. 2: 643–651. 10.1152/japplphysiol.90525.2008. 18583380

[ejsc70044-bib-0026] Ganio, M. S. , E. C. Johnson , J. F. Klau , et al. 2009. “Effect of Ambient Temperature on Caffeine Ergogenicity During Endurance Exercise.” European Journal of Applied Physiology 111, no. 6: 1135–1146. 10.1007/s00421-010-1734-x. 21120518

[ejsc70044-bib-0027] Ghandforoush‐Sattari, M. , S. Mashayekhi , C. V. Krishna , J. P. Thompson , and P. A. Routledge . 2010. “Pharmacokinetics of Oral Taurine in Healthy Volunteers.” Journal of Amino Acids 2010: 1–5. 10.4061/2010/346237. PMC3275936 22331997

[ejsc70044-bib-0028] Goldstein, E. , P. L. Jacobs , M. Whitehurst , T. Penhollow , and J. Antonio . 2010. “Caffeine Enhances Upper Body Strength in Resistance‐Trained Women.” Journal of the International Society of Sports Nutrition 7, no. 1: 18. 10.1186/1550-2783-7-18. 20470411 PMC2876999

[ejsc70044-bib-0029] Gonçalves, L. de S. , V. de S. Painelli , G. Yamaguchi , et al. 2017. “Dispelling the Myth That Habitual Caffeine Consumption Influences the Performance Response to Acute Caffeine Supplementation.” Journal of Applied Physiology 123, no. 1: 213–220. 10.1152/japplphysiol.00260.2017. 28495846

[ejsc70044-bib-0030] Gonzalez, A. M. , V. Guimarães , N. Figueiredo , et al. 2020. “Acute Caffeine Mouth Rinse does Not Change the Hydration Status Following a 10 Km Run in Recreationally Trained Runners.” BioMed Research International 2020: 1–5. 10.1155/2020/6598753. PMC7298264 32596348

[ejsc70044-bib-0031] Hein, T. W. , L. Belardinelli , and L. Kuo . 1999. “Adenosine A 2A Receptors Mediate Coronary Microvascular Dilation to Adenosine: Role of Nitric Oxide and ATP‐Sensitive Potassium Channels.” Journal of Pharmacology and Experimental Theraputics 291, no. 2: 655–664. 10.1016/s0022-3565(24)35152-3. 10525085

[ejsc70044-bib-0032] Hodgson, A. B. , R. K. Randell , and A. E. Jeukendrup . 2013. “The Metabolic and Performance Effects of Caffeine Compared to Coffee During Endurance Exercise.” PLoS One 8, no. 4: e59561. 10.1371/journal.pone.0059561. 23573201 PMC3616086

[ejsc70044-bib-0033] Hoyte, C. O. , D. Albert , and K. J. Heard . 2013. “The Use of Energy Drinks, Dietary Supplements, and Prescription Medications by United States College Students to Enhance Athletic Performance.” Journal of Community Health 38, no. 3: 575–580. 10.1007/s10900-013-9653-5. 23371823

[ejsc70044-bib-0034] Hunt, L. A. , L. Hospers , J. W. Smallcombe , Y. Mavros , and O. Jay . 2021. “Caffeine Alters Thermoregulatory Responses to Exercise in the Heat Only in Caffeine‐Habituated Individuals: A Double‐Blind Placebo‐Controlled Trial.” Journal of Applied Physiology 131, no. 4: 1300–1310. 10.1152/japplphysiol.00172.2021. 34435513

[ejsc70044-bib-0035] Jeffries, O. , M. Goldsmith , and M. Waldron . 2018. “l‐Menthol Mouth Rinse or Ice Slurry Ingestion During the Latter Stages of Exercise in the Heat Provide a Novel Stimulus to Enhance Performance Despite Elevation in Mean Body Temperature.” European Journal of Applied Physiology 118, no. 11: 2435–2442. 10.1007/s00421-018-3970-4. 30128853 PMC6182327

[ejsc70044-bib-0036] Jensen, B. N. 2000. “Accuracy of Digital Tympanic, Oral, Axillary, and Rectal Thermometers Compared With Standard Rectal Mercury Thermometers.” European Journal of Surgery 166, no. 11: 848–851. 10.1080/110241500447218. 11097149

[ejsc70044-bib-0037] Jeukendrup, A. E. , and G. A. Wallis . 2005. “Measurement of Substrate Oxidation During Exercise by Means of Gas Exchange Measurements.” Supplement, International Journal of Sports Medicine 26, no. 1: S28–S37. 10.1055/s-2004-830512. 15702454

[ejsc70044-bib-0038] John, K. , S. Kathuria , J. Peel , et al. 2024. “Caffeine Ingestion Compromises Thermoregulation and Does Not Improve Cycling Time to Exhaustion in the Heat Amongst Males.” European Journal of Applied Physiology 124, no. 8: 2489–2502. 10.1007/s00421-024-05460-z. 38568259 PMC11322244

[ejsc70044-bib-0039] Khayat, M. T. , and M. A. Nayeem . 2017. “The Role of Adenosine A_2A_ Receptor, CYP450s, and Ppars in the Regulation of Vascular Tone.” BioMed research international, The role of adenosine A2A receptor, CYP450s, and PPARs in the regulation of Vascular Tone 2017: 1720920. 10.1155/2017/1720920. PMC5572598 28884118

[ejsc70044-bib-0040] Mann, T. , R. P. Lamberts , and M. I. Lambert . 2013. “Methods of Prescribing Relative Exercise Intensity: Physiological and Practical Considerations.” Sports Medicine 43, no. 7: 613–625. 10.1007/s40279-013-0045-x. 23620244

[ejsc70044-bib-0041] Maughan, R. J. , L. M. Burke , J. Dvorak , et al. 2018. “IOC Consensus Statement: Dietary Supplements and the High‐Performance Athlete.” International Journal of Sport Nutrition and Exercise Metabolism 28, no. 2: 104–125. 10.1123/ijsnem.2018-0020. 29589768

[ejsc70044-bib-0042] Maughan, R. J. , S. M. Shirreffs , and J. B. Leiper . 2007. “Errors in the Estimation of Hydration Status From Changes in Body Mass.” Journal of Sports Sciences 25, no. 7: 797–804. 10.1080/02640410600875143. 17454547

[ejsc70044-bib-0043] Mitchell, J. W. , E. R. Nadel , and J. A. Stolwijk . 1972. “Respiratory Weight Losses During Exercise.” Journal of Applied Physiology 32, no. 4: 474–476. 10.1152/jappl.1972.32.4.474. 5026494

[ejsc70044-bib-0044] Page, L. K. , O. Jeffries , and M. Waldron . 2019. “Acute Taurine Supplementation Enhances Thermoregulation and Endurance Cycling Performance in the Heat.” European Journal of Sport Science 19, no. 8: 1101–1109. 10.1080/17461391.2019.1578417. 30776254

[ejsc70044-bib-0045] Paluska, S. A. 2003. “Caffeine and Exercise.” Current Sports Medicine Reports 2, no. 4: 213–219. 10.1249/00149619-200308000-00008. 12834577

[ejsc70044-bib-0046] Peel, J. S. , M. A. McNarry , S. M. Heffernan , et al. 2024. “The Effect of 8‐Day Oral Taurine Supplementation on Thermoregulation During Low‐Intensity Exercise at Fixed Heat Production in Hot Conditions of Incremental Humidity.” European Journal of Applied Physiology 124, no. 9: 2561–2576. 10.1007/s00421-024-05478-3. 38582816 PMC11365861

[ejsc70044-bib-0047] Peel, J. S. , M. A. McNarry , S. M. Heffernan , V. R. Nevola , L. P. Kilduff , and M. Waldron . 2021. “The Effect of Dietary Supplements on Endurance Exercise Performance and Core Temperature in Hot Environments: A Meta‐Analysis and Meta‐Regression.” Sports Medicine 51, no. 11: 2351–2371. 10.1007/s40279-021-01500-2. 34129223 PMC8514372

[ejsc70044-bib-0048] Periard, J. D. , T. M. H. Eijsvogels , and H. A. M. Daanen . 2021. “Exercise Under Heat Stress: Thermoregulation, Hydration, Performance Implications and Mitigation Strategies.” Physiological Reviews 101, no. 4: 1873–1979. 10.1152/physrev.00038.2020. 33829868

[ejsc70044-bib-0049] Ping, W. C. , C. C. Keong , and A. Bandyopadhyay . 2010. “Effects of Acute Supplementation of Caffeine on Cardiorespiratory Responses During Endurance Running in a Hot & Humid Climate.” Indian Journal of Medical Research 132: 36–41. 20693587

[ejsc70044-bib-0050] Pitchford, N. W. , J. W. Fell , M. D. Leveritt , B. Desbrow , and C. M. Shing . 2014. “Effect of Caffeine on Cycling Time‐Trial Performance in the Heat.” Journal of Science and Medicine in Sport 17, no. 4: 445–449. 10.1016/j.jsams.2013.07.004. 23932933

[ejsc70044-bib-0051] Poole, D. C. , and A. M. Jones . 2012. “Oxygen Uptake Kinetics.” Comprehensive Physiology 2, no. 2: 933–996. 10.1002/j.2040-4603.2012.tb00419.x. 23798293

[ejsc70044-bib-0052] Powers, S. K. , S. Dodd , J. Woodyard , and M. Mangum . 1986. “Caffeine Alters Ventilatory and Gas Exchange Kinetics During Exercise.” Medicine & Science in Sports & Exercise 18, no. 1: 101–106. 10.1249/00005768-198602000-00017. 3959852

[ejsc70044-bib-0053] Ramanathan, N. L. 1964. “A New Weighting System for Mean Surface Temperature of the Human Body.” Journal of Applied Physiology 19, no. 3: 531–533. 10.1152/jappl.1964.19.3.531. 14173555

[ejsc70044-bib-0054] Ravanelli, N. , G. B. Coombs , P. Imbeault , and O. Jay . 2018. “Maximum Skin Wettedness After Aerobic Training With and Without Heat Acclimation.” Medicine & Science in Sports & Exercise 50, no. 2: 299–307. 10.1249/mss.0000000000001439. 28991042

[ejsc70044-bib-0055] Ribeiro, J. A. , and A. M. Sebastião . 2010. “Caffeine and Adenosine.” Supplement, Journal of Alzheimer's Disease: JAD 20, no. S1: S3–S15. 10.3233/jad-2010-1379. 20164566

[ejsc70044-bib-0056] Roberts, W. O. 1994. “Assessing Core Temperature in Collapsed Athletes.” Physician and Sportsmedicine 22, no. 8: 49–55. 10.1080/00913847.1994.11947680. 29272635

[ejsc70044-bib-0057] Rutherford, J. A. , L. L. Spriet , and T. Stellingwerff . 2010. “The Effect of Acute Taurine Ingestion on Endurance Performance and Metabolism in Well‐Trained Cyclists.” International Journal of Sport Nutrition and Exercise Metabolism 20, no. 4: 322–329. 10.1123/ijsnem.20.4.322. 20739720

[ejsc70044-bib-0058] Sawka, M. N. , & Young, A. J. 2006. “Physiological Systems and Their Responses to Conditions of Heat and Cold.” (January) Apps.dtic.mil.

[ejsc70044-bib-0059] Schneider, D. A. , S. E. Phillips , and S. Stoffolano . 1993. “The Simplified V‐Slope Method of Detecting the Gas Exchange Threshold.” Medicine & Science in Sports & Exercise 25, no. 10: 1180–1184. 10.1249/00005768-199310000-00015. 8231764

[ejsc70044-bib-0060] Simmonds, R. , J. Cole , J. Tallent , O. Jeffries , N. Theis , and M. Waldron . 2020. “Physiological and Thermoregulatory Effects of Oral Taurine Supplementation on Exercise Tolerance During Forced Convective Cooling.” European Journal of Sport Science 22, no. 2: 1–9. 10.1080/17461391.2020.1858175. 33357070

[ejsc70044-bib-0061] Southward, K. , K. J. Rutherfurd‐Markwick , and A. Ali . 2018. “The Effect of Acute Caffeine Ingestion on Endurance Performance: A Systematic Review and Meta–Analysis.” Sports Medicine 48, no. 8: 1913–1928. 10.1007/s40279-018-0939-8. 29876876

[ejsc70044-bib-0062] Souza, D. B. , J. Del Coso , J. Casonatto , and M. D. Polito . 2016. “Acute Effects of Caffeine‐Containing Energy Drinks on Physical Performance: A Systematic Review and Meta‐Analysis.” European Journal of Nutrition 56, no. 1: 13–27. 10.1007/s00394-016-1331-9. 27757591

[ejsc70044-bib-0063] Sun, Q. , B. Wang , Y. Li , et al. 2016. “Taurine Supplementation Lowers Blood Pressure and Improves Vascular Function in Prehypertension.” Hypertension 67, no. 3: 541–549. 10.1161/hypertensionaha.115.06624. 26781281

[ejsc70044-bib-0064] Supinski, G. S. , E. C. Deal , and S. G. Kelsen . 1984. “The Effects of Caffeine and Theophylline on Diaphragm Contractility.” American Review of Respiratory Disease 130, no. 3: 429–433. 10.1164/arrd.1984.130.3.429. 6476593

[ejsc70044-bib-0065] Suvi, S. , S. Timpmann , M. Tamm , M. Aedma , K. Kreegipuu , and V. Ööpik . 2017. “Effects of Caffeine on Endurance Capacity and Psychological State in Young Females and Males Exercising in the Heat.” Applied Physiology, Nutrition, and Metabolism = Physiologie Appliquee, Nutrition et Metabolisme 42, no. 1: 68–76. 10.1139/apnm-2016-0206. 28002684

[ejsc70044-bib-0066] Ulusoy, K. G. , E. Kaya , K. Karabacak , et al. 2017. “Taurine Relaxes Human Radial Artery Through Potassium Channel Opening Action.” Korean Journal of Physiology and Pharmacology 21, no. 6: 617. 10.4196/kjpp.2017.21.6.617. 29200904 PMC5709478

[ejsc70044-bib-0067] Waldron, M. , F. Knight , J. Tallent , S. Patterson , and O. Jeffries . 2018. “The Effects of Taurine on Repeat Sprint Cycling After Low or High Cadence Exhaustive Exercise in Females.” Amino Acids 50, no. 6: 663–669. 10.1007/s00726-018-2554-2. 29549523

[ejsc70044-bib-0068] Waldron, M. , S. D. Patterson , J. Tallent , and O. Jeffries . 2018. “The Effects of an Oral Taurine Dose and Supplementation Period on Endurance Exercise Performance in Humans: A Meta‐Analysis.” Sports Medicine 48, no. 5: 1247–1253. 10.1007/s40279-018-0896-2. 29546641

[ejsc70044-bib-0069] Warnock, R. , O. Jeffries , S. Patterson , and M. Waldron . 2017. “The Effects of Caffeine, Taurine, or Caffeine‐Taurine Coingestion on Repeat‐Sprint Cycling Performance and Physiological Responses.” International Journal of Sports Physiology and Performance 12, no. 10: 1341–1347. 10.1123/ijspp.2016-0570. 28338362

[ejsc70044-bib-0070] Yu, P. , Y. Fan , and H. Wu . 2024. “Effects of Caffeine‐Taurine Co‐Ingestion on Endurance Cycling Performance in High Temperature and Humidity Environments.” Sports Health 16, no. 5: 711–721. 10.1177/19417381241231627. 38406865 PMC11346225

